# Bispecific brain-penetrant antibodies for treatment of Alzheimer’s disease

**DOI:** 10.1016/j.tjpad.2025.100214

**Published:** 2025-05-26

**Authors:** Dag Sehlin, Greta Hultqvist, Wojciech Michno, Ximena Aguilar, Amelia D Dahlén, Enrica Cerilli, Nadja M Bucher, Sara Lopes van den Broek, Stina Syvänen

**Affiliations:** a Department of Public Health and Caring Sciences, Rudbeck Laboratory, Uppsala University, Dag Hammarskjölds väg 20, Uppsala 751 85, Sweden; b Department of Pharmacy, Uppsala University, Uppsala, Sweden; c Science for Life Laboratory, Uppsala University, Uppsala, Sweden

**Keywords:** Bispecific antibody, Immunotherapy, Alzheimer’s disease, The blood-brain barrier

## Abstract

The emerging class of bispecific antibodies represents a significant advancement in Alzheimer’s disease (AD) immunotherapy by addressing the limited brain concentrations achieved with conventional monoclonal antibodies. The majority of bispecific antibodies developed for AD treatment utilize transferrin receptor (TfR1)-mediated transcytosis to enhance blood-brain barrier (BBB) penetration, resulting in higher and more uniform brain concentrations compared to conventional antibodies. This improved delivery has demonstrated superior efficacy in reducing brain amyloid-beta (Aβ) burden. Additionally, TfR1-mediated delivery may help mitigate adverse effects such as amyloid-related imaging abnormalities (ARIA). This is likely achieved by a reduction in antibody accumulation at vascular Aβ deposits, resulting from the combined effects of lower dosing and a different brain entry route when using bispecific antibodies. Besides targeting Aβ, bispecific antibodies have been engineered to address other key pathological features of AD, including tau pathology and neuroinflammatory targets, which are critical drivers of disease progression. These antibodies also show promise in diagnostic applications, particularly as radioligands for antibody-based positron emission tomography (immunoPET), leveraging their rapid brain delivery and efficient and specific target engagement. Moreover, the principles of bispecific antibody technology have been adapted for use beyond immunotherapy. The incorporation of TfR1-binding domains into enzymes, antisense oligonucleotides, or viral vectors such as adeno-associated viruses broadens their therapeutic potential. These approaches may enable more efficient treatment strategies, not only for AD but also for other neurological disorders, by facilitating the delivery of diverse therapeutic agents across the BBB.

## 1 Introduction

Until 2023, no disease-modifying drugs were available for the growing number of Alzheimer’s disease (AD) patients. Instead, treatments focused on alleviating symptoms, primarily by enhancing synaptic signaling. This was achieved by increasing acetylcholine levels through acetylcholinesterase inhibitors, which prevent acetylcholine breakdown, or by dampening glutamate signaling via pharmacological inhibition of the N-methyl-D-aspartate (NMDA) receptor. Unfortunately, these drugs do not slow disease progression, and therefore do not offer a cure for the large AD patient population.

Approximately two decades ago, antibodies targeting amyloid-beta (Aβ) were introduced in clinical trials for AD, aiming to modify the course of the disease by clearing aggregated Aβ from the brain [1]. Initial trials failed to meet their primary endpoints related to cognitive improvement due to several reasons. The first-generation antibodies, e.g., Bapineuzumab, Solanezumab and Ponezumab, were all directed towards the physiological, monomeric form of Aβ, although some of them also bind to pathological forms found in amyloid plaques and vascular Aβ deposits – cerebral amyloid angiopathy (CAA). Nevertheless, binding to monomeric Aβ or soluble fragments of the amyloid precursor protein (APP) in the blood may have contributed to reduced antibody availability and efficacy in the brain. In addition, patients included in the first clinical trials of anti-Aβ antibodies were generally in relatively advanced stages of dementia and were selected primarily based on their symptoms and clinical history, rather than biomarker findings [2]. Subsequent studies using advanced positron emission tomography (PET) imaging, which had been developed alongside Aβ-targeting therapies, demonstrated the importance of using reliable biomarkers reflecting the pathology to be treated, for inclusion of patients, as an indication of drug target engagement and for evaluation of therapeutic effect. It has been estimated that early trials included up to 35% of patients who were “amyloid-negative” by PET, and as such, would unlikely benefit from anti-Aβ treatment [3]. Furthermore, one of the first PET imaging studies demonstrated that Bapineuzumab, although it failed to meet its primary cognitive outcome measures, was associated with modest amyloid plaque reduction after 18 months of treatment, supporting the notion that Aβ immunotherapy can successfully engage its target in the brain [4].

After the first generation failed trials, it was concluded that therapeutic Aβ antibodies should selectively target pathological, aggregated forms of Aβ, and thus avoid to interfere with the physiological functions of Aβ monomers and APP [5]. Based on these insights, second-generation antibodies like Gantenerumab, Aducanumab, Lecanemab and Donanemab, all selective for pathological forms of Aβ, were evaluated in clinical trials that enrolled only biomarker-confirmed amyloid-positive patients [[6], [7], [8], [9], [10], [11]]. While amyloid-PET has the advantage that it shows both the concentration and distribution of the target, cerebrospinal fluid (CSF) biomarkers have served as a cost-effective alternative, measuring Aβ42 or the Aβ42/40 ratio as a proxy for brain amyloid deposition. Furthermore, the patients included in these studies had less advanced AD compared to those in the earlier trials with e.g., Bapineuzumab. Aducanumab received conditional FDA approval following two phase III trials. One of the trials, EMERGE, reached statistical significance for the primary endpoint (CDR-SB) for the high dose arm, while the ENGAGE study was negative. Aducanumab never acquired full FDA approval. Instead, Lecanemab was the first antibody that became widely available to patients in 2023, followed by Donanemab less than a year later. Lecanemab targets large soluble aggregates of Aβ, known as “protofibrils”, as well as insoluble Aβ plaques, while Donanemab specifically targets N-terminally truncated pyroglutamate-modified Aβ aggregates, primarily found in parenchymal Aβ plaques. All second-generation Aβ antibodies effectively reduce brain amyloid levels, with most patients becoming amyloid-PET negative within 18 months of treatment initiation. Nevertheless, treated patients still experience cognitive decline, albeit at a 25-40% slower rate than placebo-treated individuals.

Criticism surrounding these antibodies includes their high treatment cost relative to modest clinical benefits. Moreover, treatment is associated with adverse effects collectively referred to as amyloid related imaging abnormalities (ARIA), which are subdivided into ARIA-E (edema or effusion) and ARIA-H (hemorrhagic events). ARIA-E likely reflects an inflammatory response and increased vascular permeability associated with amyloid clearance. In contrast, ARIA-H is more directly linked to CAA, where vascular Aβ deposition compromises vessel integrity. ARIA-H and has been reported to a higher degree than ARIA-E also among patients included in the placebo groups. In addition, antibody interactions with Aβ deposits in the brain vasculature, particularly in the meninges and perivascular spaces of cerebral arteries, may further contribute to the development of ARIA-H. ARIA-H typically manifests as asymptomatic microhemorrhages or superficial siderosis, although severe case, including cases of fatal macrohemorrhages, have been reported. The ARIA risk contributed to the European Medicines Agency's (EMA) decision to approve Lecanemab for treatment only in patients that are not Apolipoprotein E4 (ApoEε4) homozygotes as these individuals are more likely to have CAA and are at increased risk of developing both ARIA-H and ARIA-E. Although Lecanemab seems to bind somewhat less to CAA compared to Aducanumab [12], which could be a reason for a lower incidence of ARIA-E. However, all Aβ antibodies have caused some degree of increased risk of ARIA-E and ARIA-H compared to placebo treatment.

These challenges, along with the large doses needed to achieve effective brain concentrations, have prompted a shift towards developing antibodies engineered for higher brain penetration and more uniform brain distribution. This review will discuss the current status of bispecific antibodies utilizing the transferrin receptor 1 (TfR1) to enhance brain delivery.

### 1.1 The transferrin receptor 1 and the blood-brain barrier

Antibodies are large molecules, approximately 150 kDa for immunoglobulin G (IgG), and therefore have a very limited ability to cross the tightly connected endothelial cells of the blood-brain barrier (BBB). While some studies suggest that BBB dysfunction may occur in AD, the available evidence indicates that this potential disruption is not sufficient to allow efficient antibody delivery to the brain [[13], [14], [15]]. Further, there is ongoing debate about whether antibodies cross the BBB directly or enter the brain primarily through a perivascular route [[16], [17], [18], [19]]. Regardless of the route of entry, clinical studies of Aβ-targeting antibodies suggest that, when administered at high doses over an extended period, they can penetrate deeper brain regions to some extent and help reduce brain Aβ burden.

**Fig. 1:**
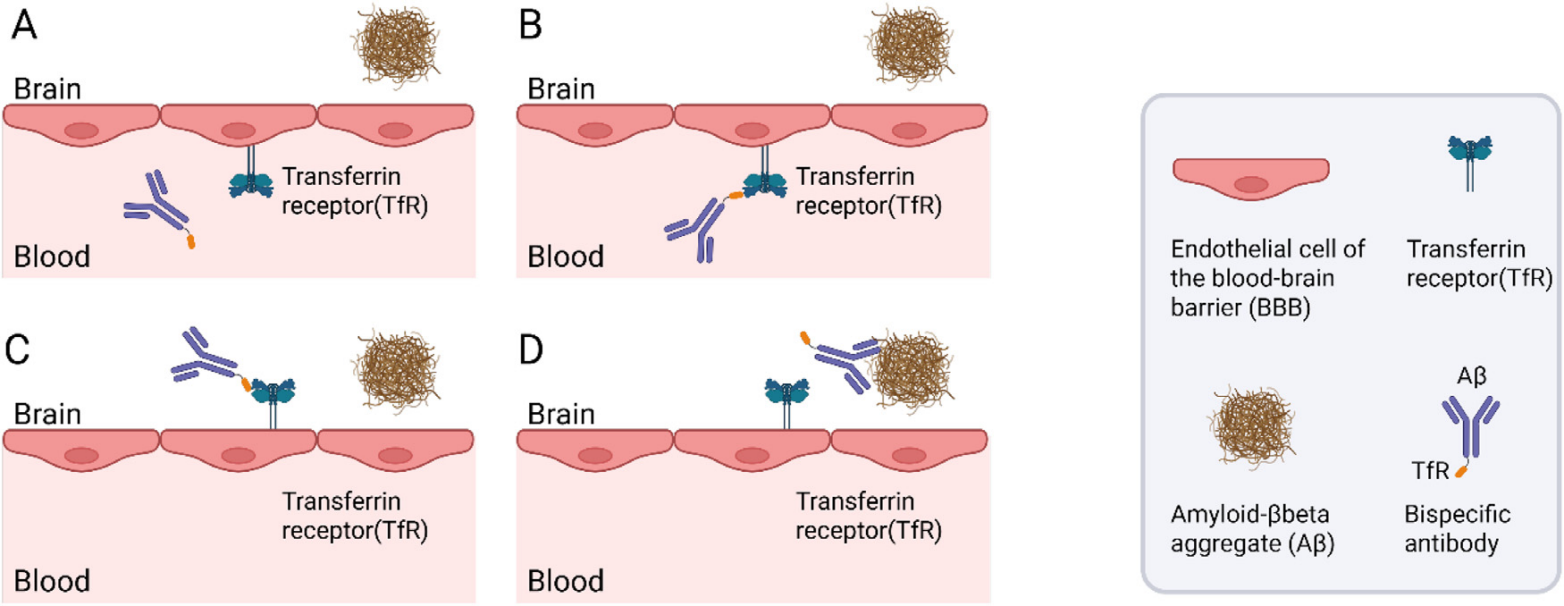
Schematic illustration of transferrin receptor (TfR)-mediated transport across the blood-brain barrier (BBB). **A.** A bispecific antibody, capable of binding both the TfR and amyloid-beta (Aβ), is administered and circulates in the brain vasculature. **B.** The bispecific antibody binds to TfR on the luminal surface of the endothelial cells of BBB. **C.** The TfR facilitates transcytosis, transporting the antibody across the BBB. **D.** Once in the brain parenchyma, the antibody binds to Aβ aggregates.

The TfR1 expressed at the blood-brain barrier (BBB) has the endogenous function of transporting iron bound to transferrin into the brain. It has been known since the 1990s that the TfR1 can be utilized to enhance the delivery of protein drugs to the brain if the drug is modified to include a TfR1-binding domain (Fig. 1). However, it was not until two decades later that substantial research efforts were directed toward understanding how to optimize the use of the TfR1 for brain drug delivery. In seminal papers published in 2011, researchers at Genentech demonstrated that moderate affinity for the TfR1 appears to be beneficial for optimized brain delivery [[20], [21], [22]]. Subsequent studies indicated that monovalent binding to the TfR1 increases delivery efficiency compared to bivalent binding [[23], [24], [25], [26]]. However, some studies suggest that total binding strength (avidity) is the key determinant of brain delivery. This implies that bivalent TfR-binders can achieve high brain delivery as long as the affinity of each binding domain is low to moderate [[27], [28], [29]]. The impact of affinity and valency may also depend on the dosage; low doses favor high affinity binders and may allow for bivalent binding (which results in high avidity), while low to medium TfR1 affinity and monovalent binding are important to achieve high brain concentrations at higher therapeutic doses of the bispecific antibody [20,25]. Furthermore, some studies suggest that pH-sensitive binding, characterized by lower affinity at lower pH, is preferable as it allows for the dissociation of bispecific antibodies in the low pH within the endosome during transport across the endothelial cells of the BBB [[30], [31], [32]]. The binding characteristics should also be optimized to match the primary intrabrain target. It is likely important that the affinity (or avidity) for TfR1 is lower than that for the primary brain target, such as Aβ. Otherwise, the bispecific antibody may preferentially bind to the TfR1 present on the surface of TfR1-expressing brain cells, e.g., neurons, instead of the primary target. This will reduce the antibody’s ability to interact with the therapeutic target and may consequently reduce the therapeutic effectiveness, and potentially also cause off-target side-effects. In addition, it may lead to uptake of antibody-Aβ complexes in neurons, which are not capable of Aβ degradation, but may instead be susceptible to intracellular Aβ seeding.

Besides the BBB, TfR1 is also expressed in peripheral tissues, such as red blood cells, the spleen, the respiratory system, and the bone marrow. Binding to sensitive peripheral tissues, including reticulocytes in the blood or bone marrow, can lead to side effects, partly mediated by the immune system. Additionally, interaction with peripheral tissues may induce target-mediated clearance of TfR1-targeted antibodies, reducing the effective drug concentration in the blood. As a result, the advantage of higher brain penetration compared to conventional antibodies can be offset by significantly decreased plasma exposure. Therefore, a moderate binding affinity, rather than a high affinity, is likely to both reduce peripheral toxicity and help maintain a relatively stable plasma pharmacokinetic profile.

### 1.2 Bispecific antibody formats directed towards amyloid-beta and TfR1

**Fig. 2:**
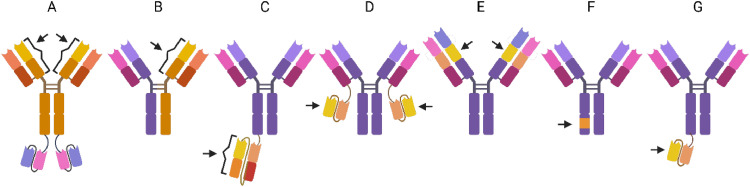
Bispecific antibody formats. A. A full-sized TfR1 antibody fused at the C-terminal ends of its heavy chains to single-chain variable fragments (scFvs) of an anti-Aβ antibody. B. A bispecific antibody comprising one heavy and light chain derived from an antibody targeting an intrabrain antigen, while the other heavy and light chain are derived from a TfR1 antibody. C. A full-sized anti-Aβ antibody fused at one of the C-terminal ends of its heavy chain to a fragment antigen-binding (Fab) region derived from a TfR1 antibody. D. A full-sized anti-Aβ antibody fused at both C-terminal ends of its light chains to scFvs derived from a TfR1 antibody. E. A tetravalent tandem bispecific antibody format with variable domains of a TfR binding antibody expressed between the constant and variable domains of a full-sized Aβ antibody. F. A bispecific antibody format (ATV) utilizing an engineered Fc domain, where one of the two heavy chains contains an amino acid sequence that binds to TfR1. G. A full-sized anti-Aβ antibody fused at one of the C-terminal ends of its heavy chain to a scFv derived from a TfR1 antibody. Blue/purple domains are directed towards Aβ, while orange/red domains, indicated by arrows, are directed towards TfR1.

Multiple research groups have developed various bispecific antibody formats that target both TfR1 and either Aβ or its production pathway. (Fig. 2). One of the earliest bispecific antibodies was constructed by fusing a full-sized TfR1 antibody, 8D3 that binds to the murine TfR1 [33], to single-chain variable fragments (scFv) of an anti-Aβ antibody (Fig. 2A) [34]. At 1 hour post-injection, this bispecific antibody demonstrated brain concentrations that were 15-fold higher than those achieved by a regular IgG. Another early example of a bispecific antibody, targeting Aβ production, comprised one heavy and light chain derived from a high-affinity antibody against the β-secretase enzyme (BACE1), combined with one heavy and light chain derived from a TfR antibody (Fig. 2B). Studies demonstrated that the bispecific antibody, anti-TfR1-anti-BACE1, achieved over 10-fold higher brain concentrations compared to the original anti-BACE1 antibody when administered to wild-type mice at a low tracer dose [20]. At a therapeutic dose of 20 mg/kg, a slightly less than 10-fold increase was observed. BACE1 is one of the enzymes that cleaves APP to form Aβ, and inhibiting this enzyme reduces Aβ production. The study also showed that the bispecific variant was more effective in reducing Aβ production in wild-type mice. This asymmetrical IgG-like format has recently been used also in a bispecific version combining different affinity variants of 8D3 with the anti-Aβ antibody 3D6 [35]. A few years after the development of the bispecific anti-TfR1-anti-BACE1 antibodies, researchers at Roche introduced the 'BrainShuttle' technology. In this approach, the original Aβ antibody, mAb31 which is the murine version on Gantenerumab, remained intact, but one or both of the heavy chains were fused at the C-terminus to a fragment antigen-binding (Fab) region derived from a TfR1 antibody [23]. For their initial proof-of-concept study, the researchers also selected the 8D3 antibody. Data from both in vitro and in vivo experiments indicated that differences in TfR1 binding mode—whether bivalent (2xFab8D3) or monovalent (1xFab8D3)—led to substantial differences in intracellular sorting within the BBB, ultimately allowing only the bispecific antibody with a single Fab8D3 to cross the BBB (Fig. 2C). The bivalent construct, on the other hand, was shown to cluster TfR1 at the cell surface and thereby induce sorting of the whole complex to the lysosome for degradation. In PS2APP transgenic mice, which express human Aβ, target engagement was increased 55-fold with the mAb31-sFab8D3 antibody compared to the original mAb31 after a single injection of 10 mg/kg. Another format, consisting of scFv8D3 fused to the C-terminal ends of the light chains of the anti-Aβ antibody mAb158 (Fig. 2D), which is the murine equivalent of Lecanemab, demonstrated an 80-fold increase in brain concentrations compared to regular mAb158 at 2 hours post-injection when administered at a tracer dose [36]. At a therapeutic dose of 10 mg/kg, this bispecific antibody achieved a 10-fold higher brain concentration [36]. It was also demonstrated that mAb158-scFv8D3 achieved the same reduction in soluble Aβ aggregates as a 10-fold higher dose of regular mAb158 following a single antibody injection [37]. A number of subsequent studies with mAb158-scFv8D3, as well as with a bispecific antibody of the same format directed towards alpha-synuclein, have confirmed the format’s ability to reduce brain Aβ and alpha-synuclein levels, respectively [[38], [39], [40]]. Furthermore, researchers at Sanofi have proposed a tetravalent tandem bispecific antibody format and demonstrated that brain concentrations increased more than 10-fold in wild-type mice compared to a conventional anti-Aβ IgG [41] (Fig. 2E). A slightly different bispecific format utilizes an engineered Fc domain, in which one of the two heavy chains contains a peptide sequence that binds to TfR1 with low affinity (Fig. 2F). This antibody format, developed by the company Denali, is referred to as Antibody Transport Vehicle (ATV) [42]. A recent study demonstrated that Aducanumab in the ATV format achieved more than a 5-fold increase in brain concentrations compared to regular Aducanumab at 24 hours post-administration of a 10 mg/kg dose [43]. Additionally, the authors introduced effector-reducing mutations (L234A/L235A, or "LALA") into the Fc domain to mitigate erythrocyte toxicity, which has been observed with some TfR1 binders due to the expression of TfR1 on blood cells. The authors concluded that the "optimal" format was to include the LALA mutation exclusively on the heavy chain containing the TfR1-binding region. This allowed for reduced toxicity while maintaining microglial activation through normal effector function, which is considered important for the removal of Aβ aggregates. Although proposed as the main mechanism for antibody induced Aβ clearance, Fc mediated microglial engagement and phagocytosis of the Aβ antibody complex has been suggested to induce proinflammatory reactions. The company Aliada (recently acquired by AbbVie) states that their construct, carrying an scFv TfR1 shuttle attached to one of the heavy chains of an Aβ antibody (Fig. 2G), mediates Aβ clearance through a non-classical phagocytosis, a phenomenon that can also be applied to neutralize cancer cells [44].

Further variations of the formats described above include modifications to the linker lengths between the Aβ and TfR1 binding domains [45], the use of scFvs of 8D3 instead of Fabs (or vice versa), and, naturally, the incorporation of different TfR1 and Aβ antibodies for the respective binding regions [[46], [47], [48]]. Furthermore, several smaller single domain antibody formats, such as variable domain of heavy-chain antibody (VHHs) and Variable new antigen Receptor (VNARs), have been developed as TfR1 binders for fusion with and delivery of therapeutic antibodies [26,30,49,50]. The BrainShuttle- and ATV-formats are asymmetrical antibodies. The production of asymmetrical antibodies requires a knob-into-hole (KiH) strategy for antibody assembly [51]. However, although KiH antibodies require a more complicated purification procedure, a recent study showed that KiH antibodies did not show a greatly reduced production yield compared to conventional IgG antibodies [46].

## 2 On-going clinical studies

The BrainShuttle format (Fig. 2C) has been further advanced with the development of the bispecific antibody Trontinemab [52], which is based on Gantenerumab, and has entered phase II clinical trials as a potential treatment for AD. Data presented at various scientific meetings highlight the superior efficacy of Trontinemab in reducing brain amyloid as measured by PET imaging, compared to the conventional approved antibodies Lecanemab and Donanemab. At a significantly lower dose of 3.6 mg/kg administered once per month, Trontinemab reduced brain amyloid to levels comparable to those in a “healthy brain” within 3-6 months in AD patients (CTAD conference, Madrid 2024). In contrast, Lecanemab and Donanemab, administered at doses of approximately 10 mg/kg, required 12 to 18 months to achieve a similar reduction [7,10]. It is important to note that no data on Trontinemab’s effects on cognitive performance have been reported thus far. Nevertheless, safety data suggest a lower incidence of ARIA-E compared to previous studies with Lecanemab and Donanemab.

There is limited information on the progress of other bispecific formats based on Aβ antibodies in clinical development. However, the company behind the approved antibody Lecanemab (Eisai/BioArctic) is actively pursuing programs to develop bispecific versions of their Aβ-antibodies. Although the exact bispecific formats have not been disclosed, representatives from BioArctic have presented preclinical data on their BrainTransporter^TM^ technology, demonstrating safety and significantly higher brain concentrations of a bispecific antibody variant compared to Lecanemab in both mice and primates (CTAD conference, Madrid 2024). Therefore, while speculative, it is likely that several other bispecific TfR1-Aβ antibodies will soon enter clinical trials.

## 3 Benefits and challenges of bispecific antibodies in comparison to conventional antibodies

**Fig. 3:**
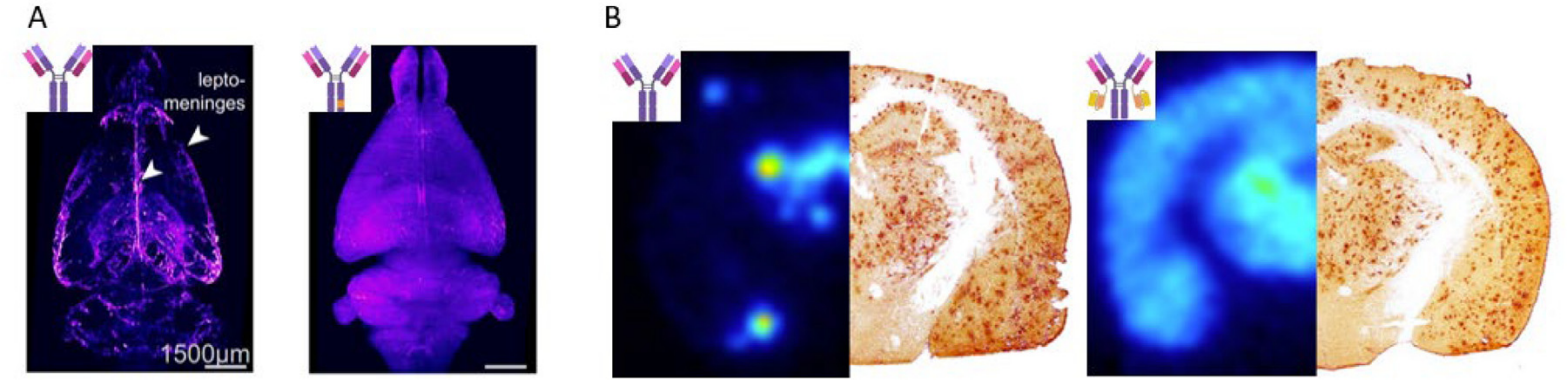
Brain distribution of regular IgG and bispecific antibodies. A Representative images from AI-segmented, 3D-reconstructed brains of wild-type mice following whole-body tissue clearing. Mice were dosed with AF647-conjugated control IgG (left) or bispecific ATV (right). One day post-administration, the bispecific ATV exhibited widespread distribution throughout the brain, whereas the control IgG was primarily localized to the brain surface, corresponding to leptomeningeal tissue and associated blood vessels, with weaker signal also observed in the lateral ventricles. Image adapted from Khoury et al., [17]. B. Brain distribution in an Alzheimer's disease (AD) mouse model dosed with radiolabeled control anti-Aβ RmAb158 (left, murine version of lecanemab) or bispecific RmAb158-scFv8D3 (right). At six days post-administration, brain distribution was visualized using *ex vivo* autoradiography and compared with Aβ40 immunostaining. RmAb158-scFv8D3 was widely distributed throughout the brain, clearly overlapping regions with Aβ pathology. In contrast, RmAb158 was mostly confined to central brain regions, likely the lateral ventricles, and appeared as hotspots, probably corresponding to large blood vessels. Image adapted from Syvänen et al., [37].

A substantial body of preclinical evidence, backed up by early clinical observations, indicates that bispecific anti-Aβ antibodies, delivered via TfR1-mediated transcytosis across the BBB, rapidly achieve higher brain concentrations than conventional antibodies and are more effective in reducing brain Aβ pathology. Moreover, these bispecific antibodies not only reach the brain in higher concentrations but also exhibit a more uniform and widespread distribution throughout the brain, with extensive retention due to interaction with parenchymal Aβ deposits [17,19,37,43,53,54] (Fig. 3). This distribution likely contributes to their enhanced therapeutic efficacy and may also reduce the risk of side effects such as ARIA-E and ARIA-H. In addition to parenchymal Aβ plaques, Aβ deposits are often present adjacent to or within the meninges and cerebral vasculature CAA, which poses significant challenges for AD immunotherapy. Conventional antibodies are believed to cross the BBB primarily through perivascular transport along large brain vessels rather than via the capillary network. This transport mechanism may lead to high local antibody concentrations at sites prone to CAA, increasing the risk of hemorrhages due to the fragility of CAA-compromised blood vessels and thereby contributing to ARIA-H. Preclinical studies suggest that bispecific antibodies are less prone to accumulate at vascular Aβ deposits [13,19], which may explain the lower incidence of ARIA-H observed in clinical trials of bispecific antibodies such as Trontinemab.

Biological drugs, including antibodies, are generally more expensive to produce than small-molecule drugs. However, bispecific antibodies may offer an economic advantage due to their potential for lower dosing requirements. Although final dosing regimens for currently approved antibodies like Lecanemab and Donanemab remain to be established, it is anticipated that lower or less frequent dosing may be recommended once patients achieve "amyloid-negativity”. At this stage, the primary goal shifts from plaque removal to preventing the accumulation of new pathology. By clearing plaques more efficiently, bispecific antibodies could enable patients to transition to maintenance dosing phases more rapidly, thereby optimizing treatment duration and cost-effectiveness.

The TfR1 is also expressed on blood cells, raising concerns about potential anemia and toxicity resulting from antibody interactions with TfR1 in the blood. Preclinical data suggest that high-affinity TfR1 binders can cause hemolysis, whereas lower-affinity binders are generally well tolerated [55,56]. This phenomenon is also likely dependent on the antibody’s architecture and effector function capacity [55]. Initial findings from the Trontinemab study indicate that anemia was not a major concern at doses up to 3.6 mg/kg (CTAD conference, Madrid 2024).

Bispecific antibodies exhibit somewhat faster clearance from the blood compared to conventional antibodies, which could potentially necessitate more frequent dosing. Antibody concentration in blood can also be affected by immune responses directed towards the therapeutic antibody, leading to the formation of neutralizing anti-drug antibodies. This phenomenon has been observed in animal studies [57,58] and is currently studied in the Trontinemab clinical trial, but has so far not been reported as a major side effect in humans. Moreover, preclinical data demonstrate prolonged brain residence times of bispecific antibodies, despite their shorter blood half-life [19,20,37,41,42,50,53,54]. Further, lower systemic concentrations may even be advantageous, as some adverse effects are likely associated with high systemic antibody levels rather than with high concentrations at the target site.

Bispecific antibodies are relatively more complex to manufacture, which could potentially offset some of the cost savings associated with their lower dosing requirements.

## 4 Bispecific antibodies directed towards other AD-related targets

In addition to the accumulation of misfolded Aβ in the AD brain, other pathological changes are also observed. For instance, the microtubule-stabilizing protein tau accumulates in the form of neurofibrillary tangles, which are considered another hallmark of AD pathology. Unlike Aβ plaques, tau inclusions are primarily intracellular, making them potentially more challenging to target. A leading strategy for tau immunotherapy is instead prevention of tau propagation and spreading in the brain, which is accomplished by targeting extracellular tau aggregates [59]. This strategy is currently evaluated both on its own and in combination with Lecanemab therapy using the anti-microtubule binding region (MTBR) tau antibody E2814 [60]. Additionally, anti- MTBR antibody BMS-986446 is being assessed independently [59]. Notably, increased concentrations of anti-tau antibodies through the use of tau-TfR1 bispecific antibodies may be crucial for this therapeutic strategy to be successful, as the spreading of tau is likely conferred primarily within the brain parenchyma. In addition, binding to diffusible tau aggregates, present at much lower concentrations than e.g. Aβ deposits, requires particularly high intrabrain concentrations of the therapeutic antibody. The use of bispecific tau antibodies may enable direct targeting of the intracellular neurofibrillary tau tangles. Since TfR1 is also expressed by neurons [17,42,61], it could potentially facilitate transport across the neuronal cell membrane to promote interaction with intracellular tau aggregates.

Another detrimental process in the AD brain is chronic neuroinflammation, characterized by the activation of glial cells, including microglia and astrocytes. This process is complex, as neuroinflammatory mechanisms may initially be beneficial by enabling microglia and astrocytes to clear misfolded and deposited proteins such as Aβ and tau. However, when neuroinflammation becomes chronic, it can itself become harmful to the brain. Various protein targets have been proposed for therapeutic intervention to dampen and resolve neuroinflammation in the AD brain. For instance, antibodies targeting the microglial protein triggering receptor on myeloid cells 2 (TREM2) have been described as a strategy to reduce neuroinflammation and Aβ plaque formation [[62], [63], [64], [65]]. In this context, some bispecific antibody formats targeting TREM2 have been developed [[66], [67], [68], [69]], although none have advanced to late-phase clinical trials. Moreover, antibodies targeting molecules other than TREM2, designed to reduce gliosis, have also been proposed as potential therapeutic agents. Such antibodies include VX15/2503 that targets semaphorin 4D [70], Ab28 that targets the LILRB2 receptor (also known as CD85d or ILT4) [71] and Daratumumab which binds to CD38 [72]. In addition, the galectin-3 binding antibody TB006 is currently in Phase II for Parkinson’s disease patents and under early clinical investigation for use in AD patients [73].

Although no bispecific construct has yet been approved for clinical use as a therapeutic for AD, similar constructs have been approved to treat patients with all forms of Mucopolysaccharidosis type II (MPS II), also known as Hunter syndrome. Patients with MPS II exhibit a deficiency in the enzyme iduronate-2-sulfatase, which is essential for the degradation of specific complex molecules, known as glycosaminoglycans (GAGs). In the absence of sufficient enzyme activity, GAGs accumulate within cells, adversely impacting tissues and organs and eventually also leading to progressive neurodegenerative symptoms. The drug Izcargo, developed by JCR Pharmaceuticals, represents the first approved biologic drug utilizing TfR1 for brain drug delivery. Izcargo comprises an anti-TfR1 antibody conjugated with the enzyme iduronate-2-sulfatase and was approved in Japan in 2021 [74].

## 5 Diagnostic bispecific antibodies

**Fig. 4:**
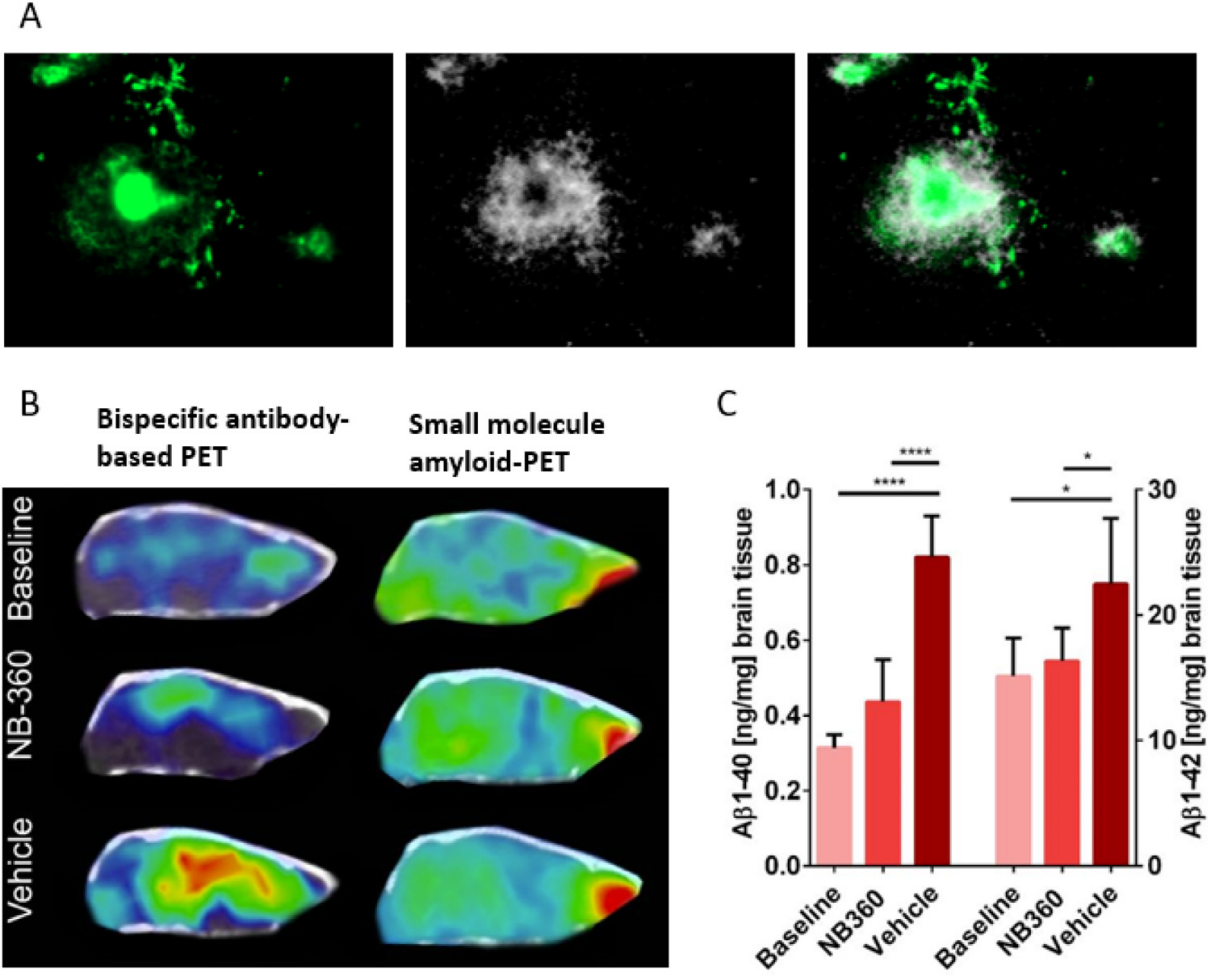
Difference between the therapeutic and the diagnostic target. A. Amyloid-β (Aβ) is visualized in green, while a bispecific version of the anti-Aβ antibody lecanemab is shown as white “grains”. The merged image (right) shows how the antibody distributes to the halo of diffuse Aβ aggregates, surrounding the amyloid core of the plaque, which remains largely inaccessible to the antibody. B. Sagittal PET images of Alzheimer’s disease model mice using a bispecific antibody-based radioligand derived from RmAb158 (the murine version of lecanemab) and the small molecule amyloid PET radioligand [¹¹C]PiB, imaged at baseline and after two months of treatment with the BACE1 inhibitor NB-360 or vehicle control. The antibody-based radioligand clearly visualizes the treatment effect, showing reduced PET signal in NB-360–treated mice compared to vehicle-treated mice, indicating lower levels of Aβ pathology. In contrast, imaging with the standard small molecule radioligand [¹¹C]PiB does not detect this reduction in Aβ levels. C. Post-mortem measurements of brain Aβ₄₀ and Aβ₄₂ concentrations in the three groups confirm the PET findings with the antibody-based radioligand, showing significantly lower Aβ levels in NB-360–treated mice compared to vehicle controls. Images B and C are adapted from Meier et al., [78].

PET imaging of amyloid has been instrumental in demonstrating that therapeutic antibodies effectively remove brain amyloid. However, the PET radioligands (radiolabeled molecules administered in minute quantities) used for this purpose bind to amyloid structures, which are primarily present in the dense core of Aβ plaques. Hence, they do not visualize soluble or diffuse aggregated forms of Aβ, which often accumulate around plaques, and represent the primary target of therapeutic Aβ antibodies (Fig. 4A). This mismatch between the therapeutic target and the diagnostic imaging target highlights a limitation in current imaging techniques. An alternative to amyloid-PET imaging could involve PET radioligands based on antibodies. However, conventional antibodies exhibit low and slow brain delivery, i.e., two characteristics incompatible with the pharmacokinetics required for PET radioligands. Bispecific antibodies, on the other hand, show rapid brain delivery and have been successfully used in preclinical models to visualize and quantify brain Aβ [54,[75], [76], [77], [78], [79], [80], [81], [82], [83], [84]] (Table 1). Notably, studies suggest that antibody-based PET imaging (immunoPET) may detect treatment effects of anti-Aβ drugs earlier than traditional amyloid-PET imaging [78,85] (Fig. 4 B&C). Bispecific antibodies have also been employed to image TREM2 in preclinical models of AD and alpha-synuclein in models of Parkinson’s disease [66,86,87].

**Table 1:** Bispecific antibodies being explored for PET-imaging purposes in Alzheimer’s disease.

Bispecific Antibody Format	TfR Binder	Primary Target	Radiolabel	References
Chemically conjugated bispecific protein	Full-length TfR antibody	Aβ	iodine-124	[80,81]
*Recombinat formats*				
Fig. 2C	Fab of TfR antibody	Aβ	zirconium-89	[75]
Fig. 2D	ScFv of TfR antibody	Aβ	iodine-124	[36,78,79,93]
Fig. 2F	TfR binding domain inserted into the Fc-region	TREM2	copper-64	[86]

Fab, fragment antigen-binding; ScFv, single-chain variable fragment; Fc, Fragment crystallizable; Aβ, amyloid-beta; TREM2, microglial protein triggering receptor on myeloid cells 2

Despite their faster clearance from the bloodstream compared to conventional antibodies, bispecific antibodies still exhibit elimination half-lives of several hours [19,53]. This delayed clearance impacts the ability to achieve optimal imaging contrast and necessitates radiolabeling with radionuclides that have longer decay half-lives, such as iodine-124 and zirconium-89, which have half-lives of approximately four days [75,80]. More recently, copper-64, with a half-life of 13 hours, has also been used for radiolabeling antibodies, including bispecific antibodies, for brain PET imaging [86,[88], [89], [90]]. Additionally, the single-photon emission computed tomography (SPECT)-compatible radionuclides indium-111 (half-life: 2.8 days) and iodine-125 (half-life: approximately two months) have been used to radiolabel bispecific antibodies for Aβ imaging [19,91]. Unfortunately, these radionuclides are less clinically compatible than carbon-11 and fluorine-18, which are commonly used to label amyloid-PET radioligands due to their short half-lives, reducing the radiation burden for patients. However, the half-life of fluorine-18 is too short for antibody ligands [83]. To address this issue, pre-targeting strategies have been proposed [92]. In this approach, the administered bispecific antibody is "tagged" but not radiolabeled. The antibody is given time to accumulate at the target site in the brain while being cleared from the peripheral circulation. After a few days, once high concentrations at the target site and low unbound concentrations in the brain and blood have been achieved, a small molecule carrying the radioactive label is administered. This small molecule is designed to bind specifically to the tag on the bispecific antibody, thereby accumulating at the target site. Whether this strategy is a viable approach for immunoPET imaging of brain targets still remains to be demonstrated in vivo.

## 6 Future

This review has focused on bispecific antibodies; however, receptor-mediated transcytosis for delivering other cargo molecules also presents significant therapeutic potential. For instance, as demonstrated by the approved drug Izcargo, TfR1-binding domains can be fused to enzymes [24,74,94,95]. Neprilysin, the primary Aβ-degrading enzyme, has its activity enhanced by the neuropeptide somatostatin. Both neprilysin and somatostatin have been successfully fused to TfR1-binding scFv8D3 domains, resulting in increased Aβ degradation in mouse models with Aβ pathology [[96], [97], [98]]. Another promising treatment strategy involves the use of antisense oligonucleotides (ASOs), which target messenger RNA, leading to its degradation or preventing its translation into proteins. ASOs have been applied to lower brain levels of Aβ and tau. Furthermore, enhancing the delivery of ASOs into the brain using TfR1-mediated transport can lead to more efficient and effective therapies [99,100].

Additionally, viral vectors such as adeno-associated viruses (AAVs) can be utilized to introduce genes encoding therapeutic proteins—such as enzymes or antibodies—into brain cells. This approach has been described as a promising strategy for treating AD and other brain disorders. While some studies suggest that AAVs can cross the BBB independently, their brain entry could likely be further improved via TfR1-mediated transport [101]. Notably, the expression of TfR1 on neuronal cells, in addition to endothelial cells, may offer another advantage by facilitating broader and more targeted delivery within the brain.

Finally, receptors other than TfR1 could also potentially be utilized for receptor-mediated transcytosis. The insulin receptor has been explored as a potential "shuttle" since the 1990s. More recently, the CD98 heavy chain (CD98hc) has been identified as an efficient receptor for this purpose, with a transport rate across the BBB that is slower than that of TfR1 but with prolonged residence time in brain, potentially offering unique advantages for specific therapeutic applications [17,[102], [103], [104], [105]]. In addition, novel strategies are being developed to extend the brain residence time of protein-based therapeutics. One approach involves recombinantly attaching an additional high-affinity antibody domain that targets an abundant brain protein, such as myelin oligodendrocyte glycoprotein (MOG). This enables the antibody to be retained in the brain for a longer duration, enhancing its therapeutic effect by prolonging drug exposure at the target site [106].

## Declaration of generative AI and AI-assisted technologies in the writing process

In the preparation of this document, AI-assisted technologies (ChatGPT) were used exclusively for language editing and grammar correction. No content was generated by AI. All intellectual contributions, analysis, and interpretations are solely those of the authors.

## CRediT authorship contribution statement

**Dag Sehlin:** Writing – review & editing, Writing – original draft, Funding acquisition, Conceptualization. **Greta Hultqvist:** Writing – review & editing, Writing – original draft, Conceptualization. **Wojciech Michno:** Writing – review & editing. **Ximena Aguilar:** Writing – review & editing. **Amelia D Dahlén:** Writing – review & editing. **Enrica Cerilli:** Writing – review & editing. **Nadja M Bucher:** Writing – review & editing. **Sara Lopes van den Broek:** Writing – review & editing. **Stina Syvänen:** Writing – review & editing, Writing – original draft, Project administration, Funding acquisition, Conceptualization.

## Declaration of competing interest

The authors declare the following financial interests/personal relationships which may be considered as potential competing interests:

Stina Syvanen reports financial support was provided by Swedish Research Council. Stina Syvanen reports financial support was provided by Swedish Brain Foundation (Hjärnfonden). Stina Syvanen reports financial support was provided by Swedish Alzheimer Foundation (Alzheimerfonden). Dag Sehlin reports financial support was provided by Swedish Research Council. If there are other authors, they declare that they have no known competing financial interests or personal relationships that could have appeared to influence the work reported in this paper.
